# Similar cardiometabolic effects of high- and moderate-intensity training among apparently healthy inactive adults: a randomized clinical trial

**DOI:** 10.1186/s12967-017-1216-6

**Published:** 2017-05-30

**Authors:** Robinson Ramírez-Vélez, Alejandra Tordecilla-Sanders, Luis Andrés Téllez-T, Diana Camelo-Prieto, Paula Andrea Hernández-Quiñonez, Jorge Enrique Correa-Bautista, Antonio Garcia-Hermoso, Rodrigo Ramirez-Campillo, Mikel Izquierdo

**Affiliations:** 1 Centro de Estudios para la Medición de la Actividad Física « CEMA», Escuela de Medicina y Ciencias de la Salud, Universidad del Rosario, Bogotá D.C, Colombia; 2 Grupo GICAEDS, Facultad de Cultura Física, Deporte y Recreación, Universidad Santo Tomás, Bogotá D.C, Colombia; 3 Laboratorio de Ciencias de la Actividad Física, el Deporte y la Salud, Universidad de Santiago de Chile, USACH, Santiago, Chile; 4 Departamento de Ciencias de la Actividad Física, Universidad de Los Lagos, Osorno, Chile; 5 Núcleo de Investigación en Salud, Actividad Física y Deporte; Laboratorio de Medición y Evaluación Deportiva, Universidad de Los Lagos, Osorno, Chile; 6 Unidad de Fisiología Integrativa, Laboratorio del Ciencias del Ejercicio, Clínica MEDS, Santiago, Chile; 7 Department of Health Sciences, Public University of Navarre, CIBER de Fragilidad y Envejecimiento Saludable (CB16/10/00315), Pamplona, Navarre Spain

**Keywords:** Randomised controlled trial, Exercise training, Metabolic syndrome, Intensity

## Abstract

**Background:**

Metabolic syndrome (MetS) increases the risk of morbidity and mortality from cardiovascular disease, and exercise training is an important factor in the treatment and prevention of the clinical components of MetS.

**Objective:**

The aim was to compare the effects of high-intensity interval training and steady-state moderate-intensity training on clinical components of MetS in healthy physically inactive adults.

**Methods:**

Twenty adults were randomly allocated to receive either moderate-intensity continuous training [MCT group; 60–80% heart rate reserve (HRR)] or high-intensity interval training (HIT group; 4 × 4 min at 85–95% peak HRR interspersed with 4 min of active rest at 65% peak HRR). We used the revised International Diabetes Federation criteria for MetS. A MetS *Z*-score was calculated for each individual and each component of the MetS.

**Results:**

In intent-to-treat analyses, the changes in MetS *Z*-score were 1.546 (1.575) in the MCT group and −1.249 (1.629) in the HIT group (between-groups difference, P =  0.001). The average number of cardiometabolic risk factors changed in the MCT group (−0.133, P = 0.040) but not in the HIT group (0.018, P = 0.294), with no difference between groups (P = 0.277).

**Conclusion:**

Among apparently healthy physically inactive adults, HIT and MCT offer similar cardiometabolic protection against single MetS risk factors but differ in their effect on average risk factors per subject.

*Trial registration* ClinicalTrials.gov NCT02738385 registered on March 23, 2016

## Background

Disorders of the metabolic system have a key pathophysiological role in the early stages of excess adiposity, elevated blood pressure, insulin resistance, abnormal glucose metabolism and dyslipidemia [elevated triglyceride levels and low-density lipoprotein cholesterol (LDL-c), and reduced high-density lipoprotein cholesterol (HDL-c)], producing coronary vasoconstriction, increasing cardiac oxygen consumption and leading to fatal events [1, 2]. This cluster of findings is recognized as metabolic syndrome (MetS) [3] and strongly predicts the risk of developing type 2 diabetes, hypertension and cardiovascular disease (CVD), which remains the leading cause of death worldwide [4–8].

Recently, Barceló [9] estimated that the number of CVD deaths in Latin America will increase by more than 60% between 2000 and 2020, while CVD deaths will increase by only 5% in high-income countries during the same period. The findings of the INTERHEART case–control study in Latin America showed that abdominal obesity, dyslipidemia, and hypertension were associated with high population-attributable risks of 48.5, 40.8, and 32.9%, respectively [10]. In the same retrospective study, daily consumption of fruits or vegetables and regular PA reduced the risk of acute myocardial infarction. Therefore, interventions aimed at the reduction of modifiable risk factors are thought to be the most effective way to prevent the onset of MetS and potentially CVD in Latin America.

On the other hand, MetS is determined by genetic predisposition as well as environmental factors that may promote its development, such as low levels of physical activity (PA), large volumes quantities of sedentary time (sitting), and poor eating habits [5, 8]. The adoption and maintenance of PA are critical foci in the metabolic health management and overall health of individuals with potential medical risks, including acute complications such as cardiac events, hypoglycemia, and hyperglycemia. Strong evidence shows that physical inactivity (<150 min week^−1^ of moderate-intensity PA or 75 min week^−1^ of vigorous-intensity PA) are jointly associated with increased cardiometabolic morbidity and mortality in a dose-dependent manner [11, 12]. Currently, physical inactivity is the fourth leading risk factor for global mortality and is comparable in that respect to smoking and obesity, accounting for 6% of all deaths [13]. Experimental studies indicate that physical inactivity and sedentary time result in alterations in cardiovascular [14] and metabolic biomarkers [15, 16].

Systematic reviews [17–19] have found that physically inactive adults who participate in supervised interval training in clinical settings improve their exercise capacity, quality of life, maximal oxygen consumption (VO_2_max) and metabolic control. A growing body of evidence has demonstrated comparable or greater improvements in cardiovascular function using low-volume high-intensity training (HIT) compared to traditional moderate-intensity continuous training (MCT) [17, 18, 20, 21]. Furthermore, participation in HIT reduces risk factors that are associated with MetS, bringing improvement in features such as the oxidative metabolism–dependent energy system, metabolic capacity, qualitative profile of skeletal muscle fiber type, muscle mass, and fiber diameter [2, 22–24]. In primary prevention, Pattyn et al. [2] shown that endurance training has a favourable effect on most of the cardiovascular risk factors associated with the MetS such as: a mean reduction in abdominal obesity, blood pressure decrease and a mean increase in HDL-c. In this same line, in previously clinical trials [22, 25–28] has been investigated the effect of exercise in different populations and for single cardiovascular risk factors, but none have specifically focused on the insufficient PA and the concomitant effect of HIT on all associated cardiovascular risk factors. However, few randomized trials have directly evaluated the effects of MCT or HIT on cardiometabolic health among inactive adults [2, 25–28].

Although the epidemiologic transition and epidemic of CVD have been well documented in Latin Americans [29–31], relatively little research on their PA [32–34] and physical fitness exists. Moreover, Latin American countries [6, 7] have a similar or even greater prevalence of MetS among adults than developed countries [8]. In this context, ethnicity and age has been associated with the development of MetS specially in Hispanic population [1, 3, 6, 7]. According to the definition of the National Cholesterol Education Program–Adult Treatment Panel III of the United States, the prevalence of MetS in adults was: 32% in Hispanic Americans; 22% in African Americans; and 24% in European Americans [3]. In Colombia, Martínez-Torres et al. [32] reported the predisposing factors for having a MetS included: being male, over 25 years old and overweight or obese, all of them related to metabolic disorders as previously described in apparently healthy women [31]. In addition, the public policy recommendations also highlight the need for healthy adults to have an activity plan that integrates preventative and therapy recommendations [40–42]. For this reason, a randomized clinical trial (RCT) comparing different intensities of exercise training in adults with insufficient PA with a large age range and different ethnic groups are clinically relevant because it can provide evidence for a precise [21, 35–37], prescribed intensity of exercise training to achieve optimal outcomes in this population [40–42].

Therefore, the purpose of this RCT was to compare the effects of MCT and HIT on the risk factors for MetS among apparently healthy physically inactive adults. We hypothesized that HIT and MCT would induce similar reductions in the risk factors for MetS and similar increases in exercise capacity when training frequency and session duration were equal in both types of training.

## Methods

### Study design and setting

The High Interval Intensity Training and ideal cardiovascular Heart Study (HIIT-Heart Study) was an RCT (ClinicalTrials.gov ID: NCT02738385) that included physically inactive Colombian adults who were randomly allocated to either an MCT group or an HIT group. The study was performed in accordance with the Declaration of Helsinki [38] and was approved by the local office of the Medical Research Ethics Committee at the University of Santo Tomás (ID 27-0500-2015). Cardiometabolic health parameters and physical fitness outcomes were assessed at baseline and 12 weeks later. We provide an overview of the methods per the Consolidated Standards of Reporting Trials (CONSORT) checklist [39].

### Participants and recruitment

This RCT was conducted at the University of Rosario and the University of Santo Tomás (Bogota, Colombia) from February 2015 to May 2016. Participants aged 18–45 years who were inactive and had a body mass index (BMI) ≥18 and ≤30 kg/m^2^ and who were willing and almost immediately available to participate in the study were recruited from the Centre of Studies in Physical Activity Measurements (*in Spanish*, CEMA) via posted study recruitment flyers at community centers, study recruitment announcements at the CEMA, and word of mouth. Individuals with a history of a medical condition identified by the American Heart Association (AHA) as an absolute contraindication to exercise testing were excluded from this study [40]. We have recently published a complete description of the HIIT-Heart Study design, methods, and primary outcomes for our current cohort [21]. Participants were required to sign a written informed consent form.

### Blinding and randomization

Random allocation into the two study groups was performed by the CEMA at the University of Rosario in Bogotá, Colombia using block randomization with a block size of four. As each consecutive participant entered this RCT, he/she was randomly allocated to either the MCT group or the HIT group according to a computer-generated group allocation sequence. The randomization sequence was not concealed from the investigator who was responsible for assigning participants to groups. The principal investigators and statisticians were blinded to treatment allocation throughout the trial protocol.

### Interventions

Both groups participated in the cardiometabolic program as recommended by both the American College of Sports Medicine (ACSM) [41] and the AHA [40, 42] guidelines for ideal cardiovascular health and disease reduction. At the beginning of the training protocol, we measured the participants’ weight to determine the weekly energy expenditure that was necessary to achieve their target of 12 kcal kg^−1^ week^−1^ (iso-energetic).

The MCT and HIT interventions lasted 12 weeks, with three sessions per week consisting of fast walking or running on a treadmill with the deck inclined to reach the desired intensity. HR was recorded during each session using an HR monitor (Polar Pacer, USA). In addition, Borg ratings were measured during each exercise session. An initial 2-week preparatory phase of training was performed to bring participants up to a 6 kcal kg^−1^ week^−1^ goal (~150 kcal per session or equivalent to 6 Mets), which was progressively increased by 2 kcal kg^−1^ week^−1^ until week 4 and was then maintained at 12 kcal kg^−1^ week^−1^ for weeks 5 through 12 (~300 kcal per session or equivalent to 10 Mets). The duration of each individual session depends on the number of visits required to reach the target kcal kg^−1^ week^−1^.

#### Moderate-intensity continuous training (MCT) group

Exercise training sessions were designed to elicit a response in the acceptable moderate-to-vigorous range, i.e., 55–75% heart rate reserve, and were adjusted according to ratings on the Borg scale. Each session consisted of a warm-up (5 min), followed by 15–55 min of treadmill walking/running (15–35 min during the 2-week preparatory phase) and a final relaxation/cool-down period (10 min).

#### High-intensity training (HIT) group

We calculated the training energy expenditure for participants’ age ranges to meet the consensus public health recommendations included in the HIIT-RT Study [21]. A complete description of the design and methods has been published elsewhere [21]. During the 2-week preparatory phase, subjects warmed up at 65% heart rate reserve (5 min), then performed 4  ×  4 min intervals at 60–80% heart rate reserve interspersed with 4 min of active recovery at 55% heart rate reserve. During weeks 3–12, subjects performed 4  ×  4 min intervals at 85–95% heart rate reserve (remaining in the target zone for at least 2 min) interspersed with 4 min of active recovery at 65% heart rate reserve and a cool-down (5 min), with a range of total exercise time ranging from 35 to 55 min (including warm-up and cool-down). We selected 6–12 kcal kg^−1^ week^−1^ per week because this dose of kcal/kg/week has produced changes in VO_2_max that placed about 70% of the initially sedentary population above the cut point for low fitness, as defined in by both the ACSM) [41] and the AHA [40, 42] guidelines for cardiovascular disease reduction.

Participants in both groups were supervised during each exercise training session by an investigator or research assistant. Exercise training was conducted at the “CEMA” fitness center on the campus of the University of Rosario, which contained the treadmills needed to complete the prescribed exercise programs. Each participant was instructed to inform the supervisor immediately if he or she experienced any unusual symptoms during exercise training and to consult a physician if needed. Participants were instructed to refrain from exercise training and to avoid changing their physical activity levels outside the study. All participants reported that they adhered to these instructions.

We estimated the energy expenditure during the exercise sessions by calibrating the energy expenditure to the HR during the maximal oxygen uptake tests performed at the baseline and post-intervention time points. The regression in energy expenditure was calculated for each participant according to both the HR and the number of minutes spent exercising during the training sessions. The trainers were physical therapists and physical educators with experience developing and monitoring exercise programs with clinical populations. Adherence to the exercise program was encouraged by the exercise professional who supervised each of the group sessions. To maximize adherence to the training program, the trainer supervised no more than 3–5 participants simultaneously. Although diet was not controlled, participants met with the study dietician for nutrition assessment and counselling at baseline, and an individualized iso-energetic nutrition intervention plan was developed from the baseline food intake assessment according to participant preferences. This plan was standardised at 1300–1500 kcal day^−1^ (50–55% carbohydrates, 30–35% total fat, <7% saturated fat and 15–22% protein), distributed across 3–4 meals per day.

### Data collection and outcome measures

The outcome measures were assessed at baseline and 12-week follow-up by personnel who were blinded to the treatment allocation. The data were recorded on standardized forms and entered into a secured Microsoft Excel Access database that included quality control checks (e.g., range checks, notifications of missing data).

Anthropometric and body composition variables were collected at the same time in the morning, between 7:00 a.m. and 10:00 a.m. Body weight and height were measured following standard procedures with an electronic scale (Tanita^®^ BC544, Tokyo, Japan) and a mechanical stadiometer platform (Seca^®^ 274, Hamburg, Germany), respectively. BMI was calculated as body weight in kilograms divided by the square of height in meters (kg/m^2^). Waist circumference (WC) was measured at the narrowest point between the lower costal border and the iliac crest using a tape measure (Ohaus^®^ 8004-MA, New Jersey, USA). In cases in which this point was not evident, WC was measured at the midpoint between the last rib and the iliac crest [43]. We measured each variable twice and used the average unless the first and second measures varied ≥1%. In such cases, we used the median of three measurements. In all measures, we found very good test–retest reliability [body weight (intra-class correlation, ICC = 0.983), height (ICC = 0.973), BMI (ICC 0.897), and WC (ICC = 0.967)]. The percentages of body fat mass and lean mass were obtained using the Tetrapolar Bioelectrical Impedance Analysis (BIA) system (SECA mBCA 515^®^, HANS E. RÜTH S.A, Hamburgo Alemania), with subjects standing barefoot on the metal contacts. This method was previously validated by experts in the field [44]. Our lab’s analysis showed strong agreement between the two methods as reflected in the range of BF%. This result shows that BIA and dual-energy X-ray absorptiometry are comparable methods for measuring body composition with higher or lower body fat percentages (unpublished data). Before testing, the participants were required to adhere to the following instructions from the BIA manufacturer [44]: (1) not to eat or drink within 4 h of the test, (2) not to consume caffeine or alcohol within 12 h of the test, (3) not to take diuretics within 7 days of the test, (4) not to perform physical exercise within 12 h of the test, and (5) to urinate within 30 min of the test. BIA measurements were performed at 50 kHz with a 0.8 mA sine wave constant current under standard conditions [44]. The measurement was made twice, and the average value was used. Inter-observer variability was R = 0.89. BIA has been extensively used as the gold standard against other body composition methods in subjects from the same region of origin as the current participants [44].

Blood pressure was measured using an electronic oscillometric device (Riester Ri-Champion model, Jungingen, Germany) according to the recommendations of the Association for the Advancement of Medical Instrumentation [45]. Prior to blood pressure monitoring, the accuracy of the device was tested using a standard mercury sphygmomanometer in a random subsample (n = 25) to ensure that there was no consistent difference (>10 mmHg) in blood pressure. To calculate the mean arterial pressure, the diastolic blood pressure was added and the sum was added to the systolic blood pressure. Inter-observer variability was R = 0.96.

Blood samples were collected between 5:30 and 7:00 a.m. by two experienced phlebotomists after ≥12 h of fasting. Blood samples were obtained from an antecubital vein, and analyses were subsequently completed within one day of collection. The biochemical profile included plasma lipid triglycerides, total cholesterol, HDL-c, LDL-c, and glucose (measured by enzymatic colorimetric methods). Inter-assay reproducibility (coefficient of variation) was determined via ten replicate analyses of five plasma pools over 15 days and was shown to be 2.6, 2.0, 3.2, 3.6% for triglycerides, total cholesterol, HDL-c and LDL-c, respectively, and 1.5% for serum fasting glucose. Additional outcomes in this study were participant adherence and adverse events. Total exercise time was defined as the total time spent on exercise training during the study. Data on participant adherence to the prescribed exercise training variables are expressed in the intervention section.

We used the revised International Diabetes Federation (IDF) [46] criteria for MetS: (i) increased waist circumference (males ≥94 cm and females ≥80 cm); (ii) increased triglycerides (≥150 mg/dl); (iii) reduced HDL-c (males <40 mg/dl and females <50 mg/dl); (iv) increased blood pressure (≥130 mm Hg systolic or ≥85 mm Hg diastolic); and (v) increased fasting glucose (≥100 mg/dl). To test the effects of exercise training on MetS, we used a continuous Z-score, rather than a series of dichotomous scores. This concept has been proposed by other researchers to represent and detect overall metabolic changes more accurately represent and detect overall metabolic changes for several reasons [22, 46]. Firstly, the continuous score would be more sensitive to small and large changes that do not change the IDF criteria [22]. Secondly, the continuous score would be less sensitive to small changes that occur in the vicinity of the diagnostic criteria for any one variable [22]. Thus, composite continuum score of MetS risk has been observed in several adult studies and has been demonstrated to be a good method to assess overall cardiometabolic risk [34]. The MetS Z-score was calculated from individual subject data, IDF criteria [46], and standard deviations using data from the entire subject cohort at baseline. The equation used was MetS Z-score = [(♂40 or ♀50—HDL-c)/SD multiplied by (−1)] + [(triglycerides—150)/SD] + [(fasting plasma glucose—100)/SD] + [(WC—♂94 or ♀80)/SD] + [(mean blood pressure—100)/SD].

### Statistical analysis

To retain the data of all randomly allocated participants, an intention-to-treat analysis (all randomly assigned patients) was performed. Prior to the planned statistical analyses, a preliminary analysis was conducted (*Kolmogorov*–*Smirnov* test) to confirm the normality of the data. Once it was confirmed that the sample data satisfied the normality assumption, statistical analyses relevant to our main research interest were conducted. *t*-tests for continuous variables and Chi square for categorical variables were used to investigate any possible differences in baseline characteristics and adherence between the groups. We used a generalized linear model (GLM) to analyze the influence of the different doses of exercise training on MetS components and body composition outcomes with repeated measures [2 (group) × 2 (test time)]. Inter-group differences in changes with time were tested using the impaired *t* test. Cohen’s *d* effect sizes (ES) were also calculated to determine the magnitude of the group differences. ES was classified as small, medium, and large as <0.20, 0.2–0.6 and 0.6–1.2, respectively [47]. The significance of the interactions effects between variables was tested using Spearman correlation analyses and denoted as *r*_*s*_. All reported P values were two-sided (P < 0.05). Statistical analyses were conducted using PASW Statistics 17 for Windows (SPSS, Inc., Chicago, Illinois).

## Results

**Fig. 1:**
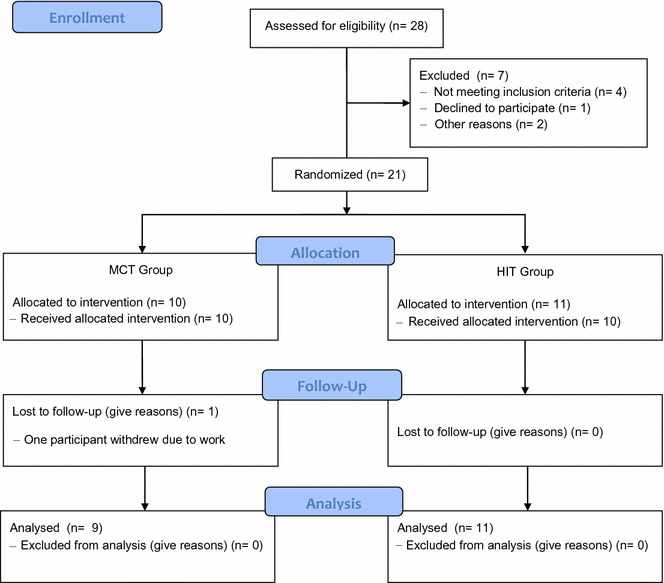
CONSORT guidelines flow diagram for enrolment and randomization HIIT-heart study

Figure 1 shows the CONSORT flowchart of the randomized clinical trial. A total of 28 potential physically inactive subjects were assessed for eligibility. Seven of them were excluded because they did not meet the inclusion criteria. Ten participants were randomly allocated to the MCT group, and 11 were allocated to the HIT group. After allocation, one participant in the MCT group withdrew for reasons unrelated to this study (lack of time due to work schedule).

The baseline characteristics of the MCT group, HIT group and total sample are outlined in Table 1. The *t*-test or Chi square indicated that no statistically significant differences in the baseline characteristics (P > 0.05) existed between the groups.

**Table 1:** Baseline participant characteristics

	Total sample (n = 20)	MCT (n = 9)	HIT (n = 11)
Sex, N (%)			
Male	8 (40.0)	5 (55.6)	3 (27.3)
Female	12 (60.0)	4 (44.4)	8 (72.7)
Age, mean (sd), years	31.8 (7.8)	31.4 (6.4)	32.1 (9.0)
Race/ethnicity, N (%)			
Black or Afro-Colombian	18 (90.0)	7 (77.7)	11 (100)
Others (indigenous)	2 (10.0)	2 (22.3)	0 (0.0)
Socioeconomic level, N (%)			
Low-mid	11 (55.0)	5 (55.5)	6 (54.5)
Mid-high	9 (45.0)	4 (45.5)	5 (45.4)
Education, N (%)			
Secondary	1 (5.0)	0 (0.0)	1 (9.1)
Technical	1 (5.0)	1 (11.1)	0 (0.0)
University	18 (90.0)	8 (88.9)	10 (90.9)
Occupation, N (%)			
Student/work	15 (80.0)	7 (77.7)	9 (81.8)
Housewife	5 (20.0)	2 (22.3)	3 (18.2)
Marital status			
Single	4 (20.0)	3 (33.3)	1 (10.0)
Married/de facto	16 (80.0)	6 (66.3)	10 (90.0)
Height, mean (sd), m	1.67 (0.06)	1.69 (0.05)	1.68 (0.09)

*BMI* body mass index

Table 2 list the effects of the exercise interventions on MetS components. For MetS Z-score a significant main effect of time was observed in MCT (P = 0.009, ES = 0.82) and HIT (P = 0.015, ES = 0.55) groups. The difference between groups was −2.795 (95% CI 1.276–4.311, P = 0.001) time × group (P = 0.001). In addition, we calculated the frequency of the MetS risk factors at each time point and the average number of MetS risk factors for each training group. The average number of cardiometabolic risk factors changed by −0.133 in the MCT group (P = 0.040); ES = 0.67 and 0.018 in the HIT group (P = 0.294); ES = 0.13 (no significant difference between groups = −0.152; P = 0.227). There was a significant increase in fasting glucose from week 0 to week 12 in the MCT group (P = 0.039); ES = 0.19 and the HIT group (P = 0.001); ES = 0.29. Although the *t*-test did not reveal significant differences between the groups [1.6 mg (95% CI −8.5–11.8; P = 0.078)], a meaningful ES increase was observed in favor of the MCT group, ES = 1.19. Mean blood pressure significantly decreased from week 0 to week 12 in the HIT group (P = 0.019 ES = 0.24), as did WC (P = 0.006 ES = 0.27) and TG (P = 0.012 ES = 0.39) in the MCT group.

**Table 2:** Intent-to-treat analysis of IDF criteria for MetS characteristics and body composition at baseline and changes after 12 weeks

	Groups				From baseline to 12-week, mean (95% CI)			MCT effect P value (ES)	HIT effect P value (ES)	Time x group P value
	Baseline		Follow-up		Whiting-group changed		Between-group difference in change			
	MCT (n = 9)	HIT (n = 11)	MCT (n = 9)	HIT (n = 11)	MCT (n = 9)	HIT (n = 11)				
MetS *Z*-score	0.668 (1.856)	−2.351 (2.384)^a^	−0.878 (1.998)	−1.102 (2.344)	1.546 (1.575)	−1.249 (1.629)	−2.795 (1.276 to 4.311)	0.009 (0.82)	0.015 (0.55)	0.001
Average risk factors per subject	0.244 (0.260)	0.164 (0.121)	0.111 (0.105)	0.182 (0.140)	−0.133 (0.200)	0.018 (0.108)	−0.152 (−0.294 to 0.004)	0.040 (0.67)	0.294 (0.14)	0.227
Waist circumference (cm)	81.9 (12.2)	75.4 (7.6)	79.5 (10.6)	75.7 (8.3)	−1.7 (3.0)	0.3 (2.6)	−2.1 (−4.7 to 0.5)	0.006 (0.27)	0.085 (0.04)	0.672
Total cholesterol (mg/dL)	170.1 (41.8)	159.4 (47.4)	153.1 (29.0)	146.6 (26.1)	−17 (37.4)	−12,7 (33.9)	−4.3 (−38.8 to 30.2)	0.102 (0.50)	0.211 (0.35)	0.452
High-density lipoprotein (mg/dL)	43.0 (14.1)	46.9 (9.6)	42.1 (9.5)	46.1 (14.1)	−0,8 (6.8)	−0.8 (10.4)	−0.1 (−8.6 to 8.5)	0.074 (0.17)	0.422 (0.07)	0.455
Triglycerides (mg/dL)	134.1 (82.2)	100.4 (36.8)	110.2 (40.1)	92.3 (45.0)	−23.8 (65.1)	−8.0 (30.9)	−15.8 (−70.5 to 38.9)	0.012 (0.39)	0.387 (0.20)	0.455
Fasting glucose (mg/dL)	82.3 (13.7)	78.3 (5.6)	85.9 (6.4)	80.2 (7.6)	3.5 (14.7)	1.9 (5.8)	1.6 (−8.5 to 11.8)	0.039 (0.19)	0.001 (0.29)	0.078
Systolic blood pressure (mmHg)	116.8 (5.1)	116.2 (6.5)	113.0 (7.6)	112.5 (9.1)	−3.8 (7.6)	−3.7 (6.5)	−0.2 (−6.8 to 6.5)	0.222 (0.63)	0.283 (0.49)	0.906
Diastolic blood pressure (mmHg)	72.3 (7.0)	71.0 (8.7)	67.8 (9.4)	67.0 (10.3)	−4.4 (8.5)	−4.0 (6.8)	−0.4 (−7.7 to 6.8)	0.274 (0.57)	0.339 (0.44)	0.960
Mean blood pressure (mmHg)	87.3 (6.0)	86.0 (7.6)	82.8 (7.8)	82.0 (9.2)	−4.4 (7.6)	−3.9 (5.4)	−0.6 (−6.8 to 5.6)	0.060 (0.68)	0.019 (0.24)	0.840
Weight (kg)	69.3 (15.3)	66.8 (10.9)	68.6 (13.5)	66.7 (10.5)	−0.6 (1.9)	−0.1 (1.6)	−0.5 (−2.2 to 1.2)	0.179 (0.05)	0.353 (0.01)	0.451
BMI (kg/m^2^)	23.6 (3.6)	25.5 (4.2)	23.4 (3.0)	24.4 (4.2)	0.2 (0.7)	1.1 (3.2)	−0.9 (−1.4 to 3.3)	0.190 (0.06)	0.130 (0.275)	0.879
Lean mass (kg)	27.4 (7.2)	30.0 (11.5)	27.4 (6.5)	31.2 (12.1)	0.0 (0.8)	1.1 (1.5)	−1.1 (−2.3 to 0.1)	0.500 (0.01)	0.010 (0.10)	0.048
Body fat (%)	24.0 (5.9)	21.1 (3.5)	24.2 (5.1)	22.0 (3.6)	0.1 (0.8)	0.9 (0.0)	0.8 (0.3 to 1.3)	0.363 (0.03)	0.032 (0.26)	0.292

*SD* data in mean, *BMI* body mass index

^a^Difference between groups at baseline

*r*_*s*_ for various anthropometric and body composition variables and the MetS *Z*-score after 12 weeks of training are presented in Table 3. Negative correlations were observed between the MetS *Z*-score, weight (*r*_*s*_ = −0.627, P = 0.011), BMI (*r*_*s*_ = −0.756, P < 0.001) and body fat (*r*_*s*_ = −0.858, P < 0.001) in the HIT group. There were no significant correlations in the MCT group.

**Table 3:** Partial correlation between MetS *Z*-score and anthropometric/body composition characteristics after 12 weeks of program training

	MCT	HIT	MCT effect (P value)	HIT effect (P value)
Weight (kg)	−0.042	−0.627	0.915	0.011
BMI (kg/m^2^)	0.001	−0.756	1.000	<0.001
Body fat (%)	0.150	−0.858	0.700	<0.001
Lean mass (kg)	−0.150	0.382	0.700	0.247

Data represent *Spearman* correlation coefficients

*BMI* body mass index

No adverse events were reported over the course of this investigation. There were differences in the total exercise time between groups (MCT, 1100 ± 258 min; HIT, 1031 ± 147 min, training days (MCT, 35.5 ± 1.3 days; HIT, 35.4 ± 0.9 days).

## Discussion

To our knowledge, this is the first RCT to compare the effects of different modes of exercise training on the clinical risk factor profile for MetS among apparently healthy physically inactive Latin American adults. The present study demonstrates that HIT was a more potent stimulus than MCT at improving a sensitive cluster of MetS risk factors, although it failed to significantly improve individual factors compared with MCT. Additionally, HIT produced stronger and moderately significant changes in MetS *Z*-score in terms of weight, BMI, and body fat.

There are divergent findings regarding MetS risk factors and HIT compared with MCT programs [11, 18, 20, 48–50]. Our study showed a higher MetS *Z*-score reduction after HIT than after MCT. The lowering of the MetS *Z*-score by supervised training is similar to what others have found in at risk patients [22, 51–53]. In addition, we found that HIT or MCT significantly reduced individual risk factors as others have found previously [22, 53]. These include reducing triglycerides levels, fat mass, abdominal obesity and mean blood pressure [2]. However, the MCT group had a higher baseline MetS *Z*-score than the HIT group, resulting in a greater improvement (ES = 0.82). In contrast to the current results, the RUSH-Study, which was performed with 81 middle-aged healthy men, showed similar positive effects on the MetS *Z*-score when HIT and MCT were compared [51]. However, in the aforementioned research, the HIT intervention included work intervals threefold longer than in the current study and thus a more prevalent aerobic component in the former, closer to MCT-induced adaptive loads [18].

Regarding unhealthy populations, studies have shown divergent findings. Confirming our results, Tjønna et al. [52] observed fewer subjects with MetS and fewer MetS risk factors in adults diagnosed with MetS after 16 weeks of HIT compared with MCT. In contrast, Johnson et al. [53] did not confirm the superiority of HIT compared with MCT in overweight and obese populations. Similarly, Earnest et al. [54] observed similar improvements in the MetS *Z*-score and the number of MetS risk factors between overweight males who participated in HIT and MCT. Due to methodological differences across studies (i.e., sex; age; initial health, weight and fitness status; prescribed medication; type and intensity of exercise, or interval duration; length of the exercise program) and the impact of such differences on outcomes [55–57], it is difficult to draw general conclusions. These and other possible factors need to be studied. The mechanism through which HIT had a greater effect than MCT on metabolic biomarkers compared to MCT is not clear. In the current study, participants in the HIT completed 4 × 4 min of exercise up to 95% of HRmax three days per week for 12 weeks, while the MCT group trained at only 55–75% of HRmax. In this context, we speculate that both training intensities might induce additive improvement in the oxidative metabolism–dependent energy system, metabolic capacity, qualitative profile of skeletal muscle fiber type, muscle mass and fiber diameter [55, 56, 58], although with potentially greater impact after HIT than after MCT. Further research is needed to reach a consensus.

No differences (time × group) were found in single MetS risk factors changes between HIT and MCT, although a significant increase in fasting glucose from baseline to post-exercise training was observed in both groups. However, levels of fasting glucose were within the healthy range. Although there are limitations to comparing Cohen’s scores in our study, the Cohen’s *d* value suggests important clinical applicability. Overall, we were unable to detect consistent superiority of HIT versus MCT programs (or vice versa) on MetS in healthy adults [18].

Furthermore, the beneficial effects of exercise on MetS *Z*-score were achieved without concomitant lean mass gainer, however, a decrease in fat mass was associated with reductions in the MetS *Z*-score (*r*_*s*_ = −0.858, P < 0.001) in the HIT group, which emphasizes meaningfulness of this change in body composition. Interestingly, the WC decreased significantly in both groups, however, *t*-test did not reveal significant differences between the groups. Changes in body composition, or more precisely, changes in abdominal obesity and fat mass seem to be an important factor when an exercise intervention for reducing CVD markers is planned. In the present study we showed that a significant reduction in MetS *Z*-score is possible also in the absence of change in lean mass.

The strengths of this study included the use of a novel Z-score to evaluate the effects of different exercise programs on the risk of MetS; this scoring method provides an increased level of sensitivity. Each subject completed at least 32 of 36 exercise sessions, and researchers supervised each session while the subjects’ HR was being monitored.

A primary limitation of this study was the lack of a true non-exercise control group. Thus, we were unable to determine causality in our interpretation of the observed exercise-induced improvements in cardiometabolic health parameters within the groups. Second, as a common tool to assess body weight and relevant body composition parameters, BIA was used in the present study. However, it is not the “gold standard” body composition measure. Due to this and other limitations (e.g., relatively small sample size; single site design), it will be important not to over-interpret the results of this RCT. Lastly, we cannot determine the directions of the associations nor causality observed in this study with absolute certainty. Future studies may consider tighter regulation of these factors to control their effects during a relatively longer intervention.

## Conclusion

HIT and MCT offer similar metabolic and cardiovascular protection against single MetS risk factors but not the average risk factors per subject. These effects could be enhanced with a reduction in fat mass that was observed only when HIT was performed. Thus, the improvement in the cardiovascular profile achieved in the present study may be an effective strategy for reduction in MetS Z-score and improving the health trajectory of physically inactive adults.

## Abbreviations

ACSM: American College of Sports Medicine
AHA: American Heart Association
BIA: bioelectrical impedance analysis
BMI: body mass index
CEMA: *in Spanish,* Centro de Estudios para la Medición de la Actividad Física
CONSORT: consolidated standards of randomized clinical trials
CVD: cardiovascular disease
ES: effect sizes
GLM: generalized linear model
HDL-c: high-density lipoprotein cholesterol
HIIT-Heart Study: high interval intensity training and ideal cardiovascular heart study
HIT: high-intensity training
HR: heart rate
HRR: heart rate reserve
ICC: intra-class correlation
IDF: International Diabetes Federation
LDL-c: low-density lipoprotein cholesterol
MCT: moderate-intensity continuous training
MetS: metabolic syndrome
PA: physical activity
RCT: randomized clinical trial
*r*_*s*_: spearman correlation
VO_2_max: maximal oxygen consumption
WC: waist circumference
