# Erratum to: Similar cardiometabolic effects of high- and moderate-intensity training among apparently healthy inactive adults: a randomized clinical trial

**DOI:** 10.1186/s12967-017-1238-0

**Published:** 2017-06-13

**Authors:** Robinson Ramírez-Vélez, Alejandra Tordecilla-Sanders, Luis Andrés Téllez-T, Diana Camelo-Prieto, Paula Andrea Hernández-Quiñonez, Jorge Enrique Correa-Bautista, Antonio Garcia-Hermoso, Rodrigo Ramirez-Campillo, Mikel Izquierdo

**Affiliations:** 1 Centro de Estudios para la Medición de la Actividad Física «CEMA», Escuela de Medicina y Ciencias de la Salud, Universidad del Rosario, Bogotá D.C, Colombia; 2 Grupo GICAEDS, Facultad de Cultura Física, Deporte y Recreación, Universidad Santo Tomás, Bogotá D.C, Colombia; 3 Laboratorio de Ciencias de la Actividad Física, el Deporte y la Salud, Universidad de Santiago de Chile, USACH, Santiago, Chile; 4 Departamento de Ciencias de la Actividad Física, Universidad de Los Lagos, Osorno, Chile; 5 Núcleo de Investigación en Salud, Actividad Física y Deporte; Laboratorio de Medición y Evaluación Deportiva, Universidad de Los Lagos, Osorno, Chile; 6 Unidad de Fisiología Integrativa, Laboratorio del Ciencias del Ejercicio, Clínica MEDS, Santiago, Chile; 7 Department of Health Sciences, Public University of Navarre, CIBER de Fragilidad y Envejecimiento Saludable (CB16/10/00315), Pamplona, Navarre Spain

## Erratum to: J Transl Med (2017) 15:118 DOI 10.1186/s12967-017-1216-6

In the original version of this article [1], published on 30 May 2017, we noticed an error in Table 2. The lean mass (kg) and fat mass (%) corrected table is included in this erratum.

**Table 2:** Lean mass (kg) and fat mass (%)

	Groups				From baseline to 12-week, mean (95% CI)			MCT effect p value (ES)	HIT effect p value (ES)	Time × group p value
	Baseline		Follow-up		Whiting-group changed		Between-group difference in change			
	MCT (n = 9)	HIT (n = 11)	MCT (n = 9)	HIT (n = 11)	MCT (n = 9)	HIT (n = 11)				
Lean mass (kg)	49.7 (9.3)	44.9 (5.8)	49.4 (8.3)	45.9 (5.9)	−0.3 (1.2)	1.1 (1.2)	−1.4 (−2.5 to −0.2)	0.237 (0.01)	0.007 (0.10)	0.156
Body fat (%)	27.4 (7.3)	31.3 (12.2)	27.4 (6.6)	30.1 (11.5)	0.0 (0.9)	−1.2 (1.5)	1.2 (0.0 to 2.4)	0.500 (0.03)	0.014 (0.26)	0.026

These changes have no material impact on the conclusions of our paper. We apologize for any inconvenience caused to our readers.
